# Case Report: Large nested variant urothelial carcinoma –invasive malignancy masquerading as low grade disease

**DOI:** 10.12688/f1000research.5966.1

**Published:** 2014-12-23

**Authors:** Andrew Keller, Ai Jye Lim, Ahmad Ali

**Affiliations:** 1 Department of Urology, Ipswich General Hospital, Ipswich, QLD, 4305, Australia; 2 Department of Anatomical Pathology, Princess Alexandra Hospital, Woolloongabba, QLD, 4102, Australia

**Keywords:** LNVUC, Urothelial carcinoma

## Abstract

**Introduction**

The large nested variant of urothelial carcinoma (LNVUC) is a newly described and rare subtype of urothelial carcinoma. It is characterised by bland cytological features and a large nested architecture similar in appearance to low grade urothelial carcinoma with an inverted growth pattern. To date only 23 cases in a single series have been described.

**Case Report**

We describe the case of a 59 year old male with LNVUC whose tumour was initially misdiagnosed as a non-invasive low grade urothelial carcinoma. At a subsequent re-resection, his tumour was correctly re-classified as LNVUC with extensive invasion of the muscularis propria. Radical cystectomy and formation of an ileal conduit was performed. His operative specimen revealed invasion of prostatic stroma and perivesical fat, with all surgical margins clear. He is currently free from clinical recurrence 12 months after his cystectomy.

**Conclusion**

LNVUC is a newly described and rare urothelial carcinoma subtype. It characteristically possesses bland cytological features and may mimic low grade urothelial cancer. Despite its bland appearance it behaves aggressively with invasion, metastasis and death being common.

## Introduction

The large nested variant of urothelial carcinoma (LNVUC) is a newly described variant of urothelial cancer (UC), with a single series of 23 cases being the only examples reported thus far ^1^. This aggressive UC variant has deceptively bland cytological features, which may confound correct tumour classification. We present the case of a 59 year old male with a large bladder tumour who was initially diagnosed histologically as non-invasive low grade UC on initial resection. At re-resection the tumour was correctly identified as LNVUC.

## Case report

A 59 year old Caucasian male was transferred to our unit from a regional hospital with a two week history of macroscopic haematuria. He sought medical attention only after he developed clot retention. He denied any previous history of haematuria or urinary problems prior to the two week period immediately before his hospital admission.

His medical history was unremarkable other than extensive carcinogen exposure, with both a 40 pack-year smoking history and significant occupational exposure, working as a fly-in, fly-out diesel fitter on a mine site.

On admission he required placement of an indwelling urinary catheter and continuous bladder irrigations. His initial serum creatinine was elevated, but soon normalised following catherisation. He was transferred to our secondary referral centre following failure of conservative therapies to control his persistent haematuria.

**Figure 1.**
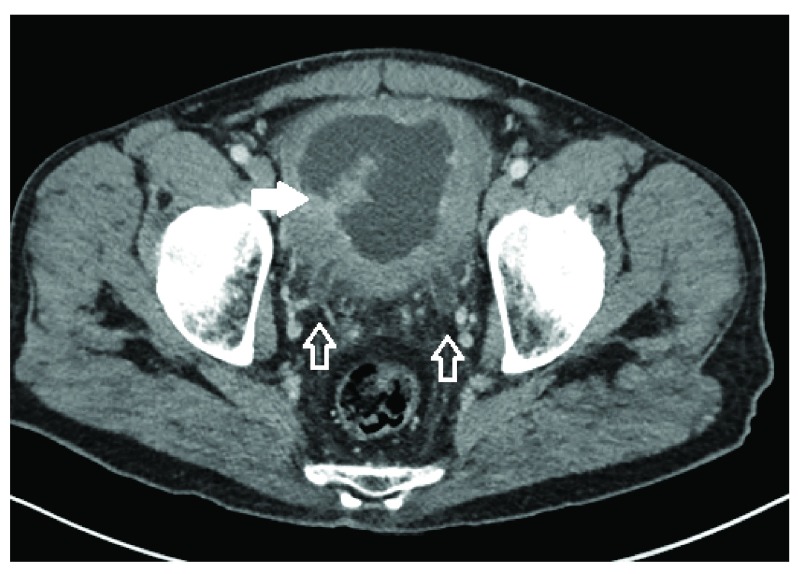
Axial contrast enhanced CT demonstrating bilateral ureteric dilation (hollow arrows) and a large enhancing intravesical mass (solid arrow).

On his arrival to our facility we arranged Computerised Tomography (CT) to assess his bladder and upper renal tracts. CT demonstrated a grossly thick walled bladder with a large enhancing intra-vesical mass, and bilateral hydroureteronephrosis ( Figure 1). His haematuria continued and he became transfusion dependant. He was taken to the operating theatre two days after his arrival for cystoscopic assessment.

**Figure 2.**
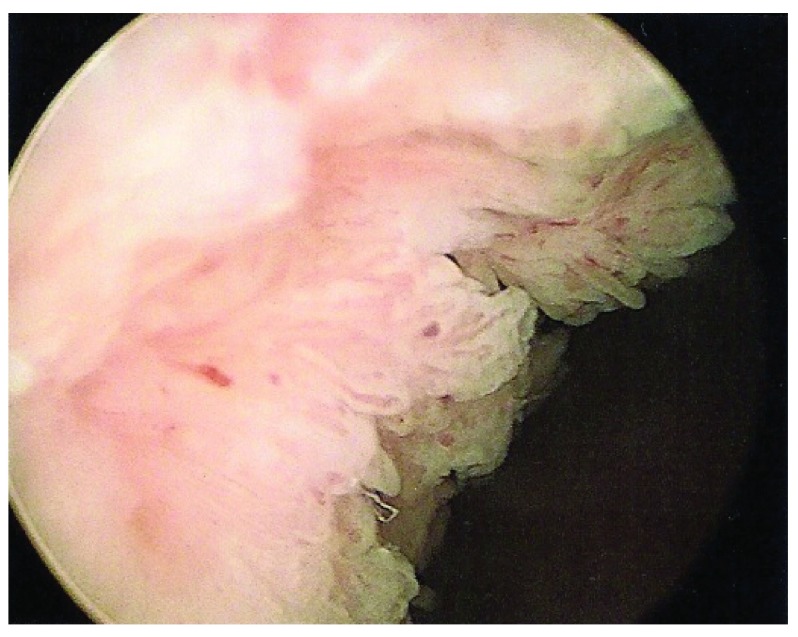
The bland appearance of the tumour at the bladder neck at his initial cystoscopy.

At cystoscopy, there was a large papillary tumour involving the prostatic urethra, the trigone, and both lateral walls of the bladder. ( Figure 2) Neither ureteric orifice was identifiable. The tumour was macroscopically resected after an extensive procedure.

Histologically the tumour was classified as a low grade urothelial carcinoma with no evidence of superficial or muscle invasion. We found this finding inconsistent with the operative and radiologic findings and repeated a cystoscopy four weeks later.

**Figure 3.**
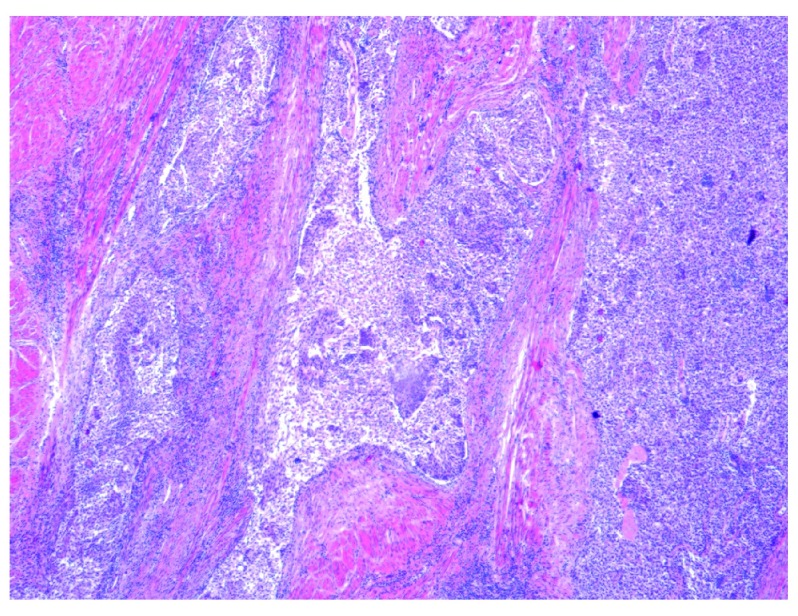
Low power view (×4 objective lens) of Hematoxylin and Eosin (H and E) stained bladder resection specimen showing the large nests of the invasive component of urothelial carcinoma with abundant interspersed stromal tissue.

**Figure 4.**
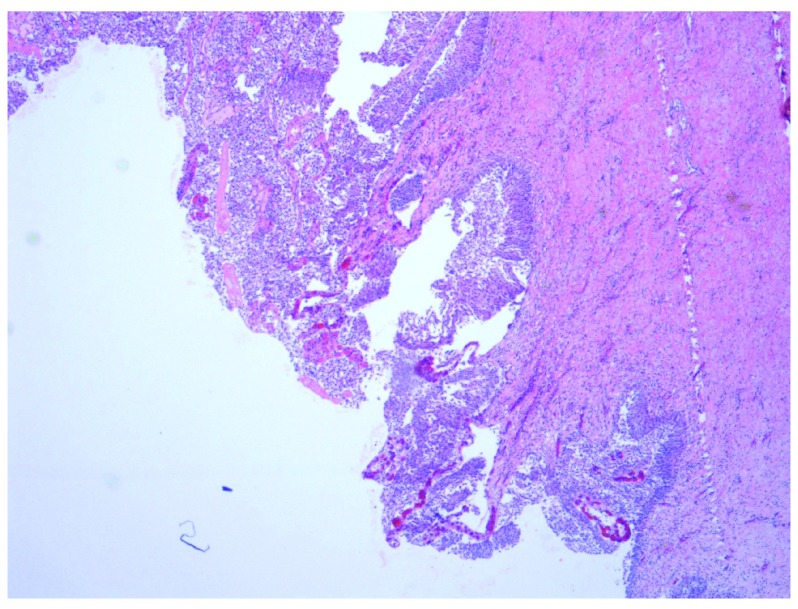
Low power view (×4 objective lens) of Hematoxylin and Eosin (H and E) stained bladder resection specimen showing the superficial component of LNVUC.

At repeat cystoscopy large volume tumour regrowth had occurred and a further 90 minute resection was performed. Tumour histology this time demonstrated invasion into the muscularis propia by a large nested variant of UC ( Figure 3) with an adjacent superficial component of low grade papillary UC ( Figure 4).

**Figure 5.**
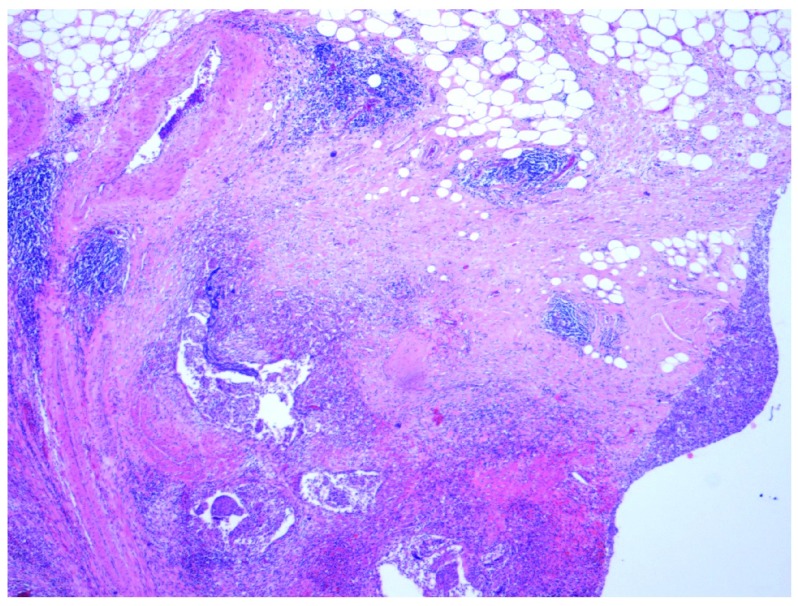
Low power view (×4 objective lens) of Hematoxylin and Eosin (H and E) stained bladder resection specimen showing focal invasion of tumour into peri-vesical fat.

**Figure 6.**
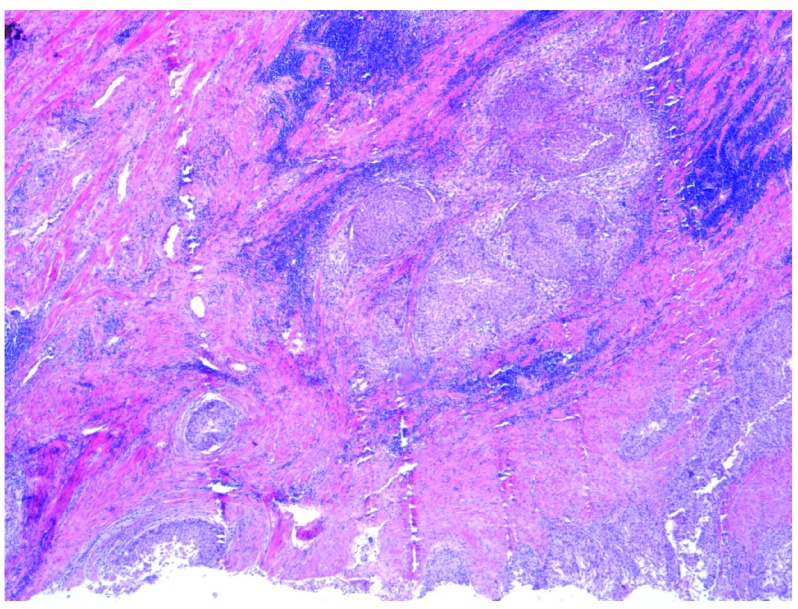
Low power view (×4 objective lens) of Hematoxylin and Eosin (H and E) stained bladder resection specimen showing tumour nests invading prostatic stromal tissue.

A staging Positron Emission Tomography (PET) CT was negative for metastatic disease and a cysto-prostatectomy and formation of an ileal conduit was performed. The operative specimen histology again revealed the large nested variant of UC with focal invasion into peri-vesical fat ( Figure 5) and the prostatic stroma ( Figure 6). A component of low grade UC was also present superfically. The tumour was clear of all operative margins. All lymph nodes sampled were negative for metastatic deposits.

The patient’s post-operative period was unremarkable and he made a swift recovery. He was discharged from hospital one week post-operatively. He was referred to medical oncology for consideration of adjuvant chemotherapy, however after discussion with oncologists the patient declined any additional treatment. He is presently twelve months post cysto-prostatectomy and he remains clinically well and free from clinical disease recurrence. We will continue to closely monitor this patient.

## Discussion

The large nested variant is a newly described subtype of UC. The first and to date only case series was published in 2011 by Cox and Epstein and describes 23 cases ^1^. They describe tumours with universally bland histologic appearances but with invasion of large nests resembling von Brunns nests into the underlying stroma. In contrast to the normal nested variant of UC, a surface papillary component is present and there is abundant fibrous stroma between individual tumour nests ^1, 2^. LNVUC is most commonly mistaken for low grade urothelial cancer with an inverted growth pattern ^2^.

LNVUC behaves aggressively, of the 17 cases with adequate follow-up in Cox and Epstein’s series, 3 had died of their disease and another two were alive but had developed metastatic spread of their cancer ^1^.

## Conclusion

The large nested variant is an extremely rare, newly described variant of UC. Our case is only the 24th described in the literature, and the first case reported since the condition was first classified in 2011. LNVUC can confound accurate diagnosis by masquerading as Von Brunn’s nests or, in our case, low grade non-invasive UC. Despite the bland macroscopic and histologic appearance of LNVUC it behaves in an aggressive manner, and should be treated the same as any invasive urothelial malignancy.

## Consent

Written informed consent for publication of their clinical details and/or clinical images was obtained from the patient.
