# Supplementary material for: Case Report: Large nested variant urothelial carcinoma –invasive malignancy masquerading as low grade disease
Source: F1000Res. 2014 Dec 23;3:314. [Version 1] doi: 10.12688/f1000research.5966.1 (PMC4314662; doi:10.12688/f1000research.5966.1)
Supplement: Peer review history

# Peer review: Referee response for version 1

**DOI:** 10.5256/f1000research.6383.r7541

**Published:** 2015-02-02

**Authors:** Daron Smith, Paula Pal

**Affiliations:** 1 Institute of Urology, University College London Hospitals NHS Trust, London, UK; 2 University College Hospital, London, UK

This case report does an excellent job of highlighting the deceptive nature of LNVUC and the clinical importance of early detection. This aggressive and rare subtype of urothelial cancer can very easily be mis-diagnosed as a low-grade urothelial tumour. The article identifies the key demographic risk factors seen in LNVUC (male, heavy smoker, age- approx 60yrs) and highlights the clinical factors that point towards an aggressive pathology (upper tract dilatation, rapid regrowth). Early recognition is vital in allowing prompt radical treatment.

In our unit we have also had a very similar case of a bladder tumour which on resection was found to contain discohesive nests of invasive urothelial carcinoma. This patient also underwent radical cystectomy and final histology confirmed T3 disease.

As LNVUC can cause a diagnostic dilemma, we would be interested to learn if any immunohistochemical studies were carried out and what these showed.

We have read this submission. We believe that we have an appropriate level of expertise to confirm that it is of an acceptable scientific standard.

# Peer review: Referee response for version 1

**DOI:** 10.5256/f1000research.6383.r7138

**Published:** 2015-02-02

**Authors:** Levent Turkeri

**Affiliations:** 1 Department of Urology, Marmara University School of Medicine, Istanbul, Turkey

This is a timely report on large nested variant of urothelial carcinoma. It may pose a diagnostic confusion since histological appearance may resemble more indolent forms of the disease although it certainly has a high invasive potential and may be associated with metastatic disease. Therefore, the pathologists must be aware of this variant when they evaluate surgical specimens and urologists must discuss with their pathologists the possibility of the presence of such a variant when they receive a report indicating low grade disease yet the clinical presentation is not compatible.

I have read this submission. I believe that I have an appropriate level of expertise to confirm that it is of an acceptable scientific standard.

# Peer review: Referee response for version 1

**DOI:** 10.5256/f1000research.6383.r7134

**Published:** 2015-01-07

**Authors:** M Hammad Ather

**Affiliations:** 1 Department of Surgery, Aga Khan University, Karachi, 74800, Pakistan

Nested variant of UC is indeed rarely reported. It is however, not certain whether the actual incidence is low or it is less frequently recognized. The estimated incidence is 0.3% ( Hong *et al., *2007).

Authors have correctly recognized the disparity in clinical parameters (Significant exposure, 40 pack year history, industrial exposure, large bladder growth and obstructive uropathy) with the pathology indicating low grade cancer (pTa LG).Most of the recognized clinical indicators of nested variant including male gender, age above 50 years, ureteral obstruction and cystoscopy showing  tumor slightly raised and erythematous or nodular were present in this case.

I have few queries on which i request the authors' to comments

1. Tumor is known to have aggressive behavior, however, growth in 4 weeks needing 90 minute resection is rather surprising.
2. Were any cold cup deep biopsies taken at the time of initial resection in view of the significant clinical findings of aggressive UC.
3. Were biopsies from the bladder neck, prostatic fossa or para collicular area taken at the time of initial resection.

I have read this submission. I believe that I have an appropriate level of expertise to confirm that it is of an acceptable scientific standard.

# Response

**Published:** 2015-01-07

**Authors:** Andrew Keller

**Affiliations:** Ipswich General Hospital, Australia, Australia

Thanks for your review.

The described pathology in this case report is actually "large nested UC" which is a distinct clinical entity to "nested UC" and one only recently described. I agree that this entity, like "nested UC" is undoubtedly more common than is documented due to under reporting.

In regards to your queries.

1. I agree that the amount of tumour re-resection needed after only a 4 week interval following a macroscopically complete resection is surprising, it certainly was for us. One explanation is that a surgical trainee and  not a consultant urologist was performing the resection, which would account for some of the additional time required.

2. No cold cup biopsies were taken at the time of the initial resection as the resection was felt to have adequately sampled the muscularis propria. Indeed, there was plentiful muscle present in the initial resection specimen histologically.

3. Cold cup biopsies were not taken from the bladder neck, prostatic fossa and paracollicular area at the time of the initial biopsies. However both bladder neck and prostatic fossa proximal to the prostatic utricle were involved with tumour at initial resection and these areas were consequently extensively resected with loop diathermy.

Thanks again for your comments and review.

Andrew Keller
